# Identification of genetic risk factors for brain amyloid deposition

**DOI:** 10.1002/alz.092672

**Published:** 2025-01-03

**Authors:** Maulikkumar P Patel, Kang‐Hsien Fan, Matthew Johnson, Arda Cetin, Muhammad Ali, Daniel Western, Menghan Liu, Priyanka Gorijala, John P. Budde, Ann D Cohen, Oscar L. Lopez, Eleanor Feingold, Cyril P Pottier, M. Ilyas Kamboh, Carlos Cruchaga

**Affiliations:** ^1^ Department of Psychiatry, Washington University, St Louis, Saint Louis, MO USA; ^2^ Neurogenomics and Informatics, Washington University, St. Louis, Saint Louis, MO USA; ^3^ Washington University in St. Louis School of Medicine, Saint Louis, MO USA; ^4^ School of Public Health, University of Pittsburgh, Pittsburgh, PA USA; ^5^ Washington University in saint Louis School of Medicine, Saint Louis, MO USA; ^6^ Department of Psychiatry, Washington University, St Louis, Saint Louis, MO USA; ^7^ Neurogenomics and Informatics, Washington University, Saint Louis, MO USA; ^8^ Department of Psychiatry, Washington University, Saint Louis, MO USA; ^9^ Department of Psychiatry, Washington University, St. Louis, MO USA; ^10^ NeuroGenomics and Informatics Center, Washington University, St. Louis, MO USA; ^11^ Washington University in St. Louis School of Medicine, St. Louis, MO USA; ^12^ NeuroGenomics & Informatics Center, Washington University School of Medicine, St. Louis, MO USA; ^13^ Department of Psychiatry, Washington University School of Medicine, St. Louis, MO USA; ^14^ Washington University School of Medicine, Saint Louis, MO USA; ^15^ University of Pittsburgh Alzheimer’s Disease Research Center (ADRC), Pittsburgh, PA USA; ^16^ University of Pittsburgh, Pittsburgh, PA USA; ^17^ Department of Psychiatry, Washington University,, Saint Louis, MO USA; ^18^ Department of Neurology, Washington University, School of Medicine, Saint Louis, MO USA; ^19^ Department of Human Genetics, University of Pittsburgh, Pittsburgh, PA USA; ^20^ Hope Center for Neurological Disorders, Washington University School of Medicine, St. Louis, MO USA; ^21^ Washington University in St. Louis, School of Medicine, St. Louis, MO USA

## Abstract

**Background:**

Amyloid PET imaging is a promising biomarker to track the accumulation of parenchymal amyloid beta (Aβ) deposits in the brain. Recent large‐scale genome‐wide association studies (GWAS) reported common risk factors associated with amyloidosis, suggesting that this endophenotype is driven by genetic variants. We hypothesized that genes with multiple variants with deleterious effect are associated with Aβ accumulation. To address this issue, we performed whole‐genome sequencing (WGS) on a large cohort of individuals with amyloid PET imaging.

**Method:**

In this study, we carried out a (GWAS) on European‐ancestry individuals with amyloid PET imaging from two cohorts [MAP (Memory & Aging Project), and Pitt (University of Pittsburgh)]. WGS was performed using Illumina Hiseq followed by GATK variant calling as well as extensive variant and ‐individual level quality check. Functional annotation was performed using snpEff and dbNFSP. Only variants predicted loss of function (stop loss/gain, splicing, and frameshift variants), defined as high effect; or with Combined Annotation Dependent Depletion score greater than 25, defined as moderate effect, were included. The amyloid PET harmonized z‐score was used as a quantitative phenotype in our SKAT‐O gene based analyses.

**Result:**

After quality control, we retained 1,228 participants (52% female). Gene‐based analyses identified a study‐wide significant association for *APOE* (p‐value = 1.82 × 10^‐09^), and eight additional genes with suggestive significance association (p‐value<7.5 × 10^‐04^) including *MPDZ*, *SEC16A*, *PLBD1*, *GLB1L2*, *KCNMB4*, *ELOVL5*, *SCART1* and *SCN7A*. According to the GTEx portal, five of these candidate genes are expressed in the brain. *MPDZ* encodes for a modular scaffold protein that was recently reported to be associated with Alzheimer’s disease in African‐Americans, supporting its involvement in amyloidosis risk (Ray et al., MedRxiv, 2023)

**Conclusion:**

We present the first time, a gene‐based association study assessing the effect of potentially deleterious variants on amyloidosis. Importantly, we show that variants located in the *APOE* gene have a strong effect on this endophenotype. Interestingly, we point out additional candidate genes such as *MPDZ* that are potentially associated with amyloidosis. Additional replication, however, is required to validate our findings.

